# Supplementary material for: Designing a Functional CNT+PB@MXene-Coated Separator for High-Capacity and Long-Life Lithium–Sulfur Batteries
Source: Membranes (Basel). 2022 Jan 23;12(2):134. doi: 10.3390/membranes12020134 (PMC8879464; doi:10.3390/membranes12020134)
Supplement: Supplementary file 1 [file membranes-12-00134-s001.zip › membranes-1535122-supplementary.pdf]

membranes

MDPI

Supplementary Materials

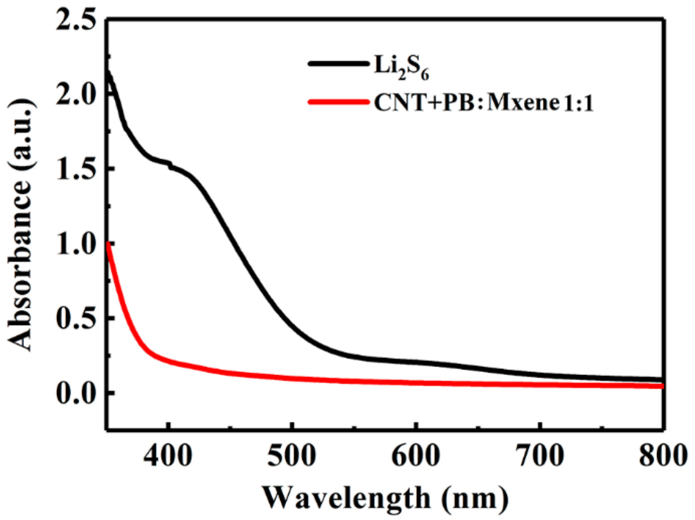

Figure S1. UV spectra of Li_2S_6 solutions before and after Li_2S_6 adsorption of CNT+PB@Mxene.

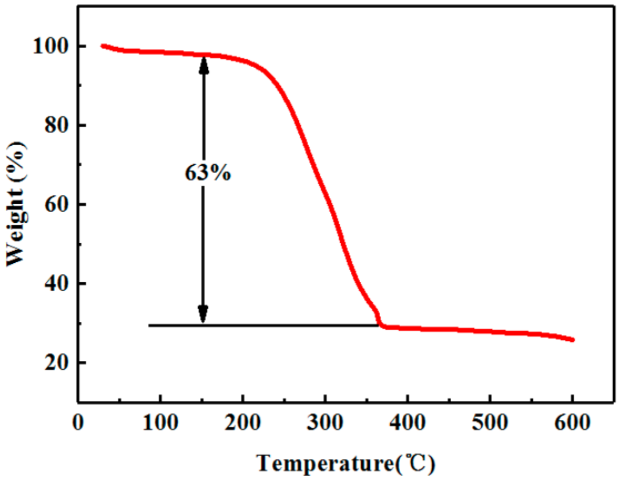

Figure S2. TGA curve in N_2 of the cathode of carbon-sulfur composite.

Figure S2 is the TGA curve tested in nitrogen at a rate of 10 ^{o}C min^{-1}, and analysis indicates the content of sulfur in carbon-sulfur composite is 63%.

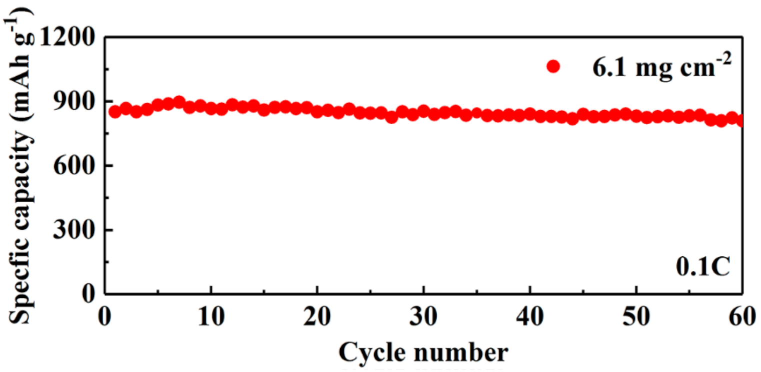

Figure S3. Long-term cycling curve of LSB with a large sulfur load of 6.1 mg cm^{-2} used CNT+PB@Mxene/PP as separator.
